# Novel Wide-Band Dielectric Imaging System Guided Lead Deployment for His Bundle Pacing: A Feasibility Study

**DOI:** 10.3389/fcvm.2021.712051

**Published:** 2021-09-03

**Authors:** Wei Hua, Xi Liu, Min Gu, Hong-xia Niu, Xuhua Chen, Min Tang, Shu Zhang

**Affiliations:** Cardiac Arrhythmia Center, Fuwai Hospital, National Center for Cardiovascular Diseases, Chinese Academy of Medical Sciences and Peking Union Medical College, Beijing, China

**Keywords:** His bundle pacing, radiation exposure, electroanatomical mapping, fluoroscopy, implantation technique

## Abstract

**Introduction:** His bundle pacing (HBP) is the most widely used physiological pacing modality, but difficulties in locating the His bundle lead to high fluoroscopic exposure. An electroanatomical mapping (EAM) system can be an efficient tool to achieve HBP implantation with near-zero fluoroscopic visualization.

**Methods:** In the study, 20 patients who had indications for pacemaker implantation were prospectively enrolled and underwent HBP implantation either with the conventional fluoroscopy approach (the standard group) or guided by a novel KODEX-EPD mapping system (the EAM-guided group). The success rate, procedural details, pacing parameters, and procedure-related complications were compared between the two groups.

**Results:** In the study, 20 consecutive patients were randomized with 10 patients in each group. HBP was successfully achieved in nine patients in the standard group and nine patients in the EAM-guided group. The procedural time was similar between the EAM-guided group vs. the standard group (85.40 ± 22.34 vs. 86.50 ± 15.05 min, *p* = 0.90). In comparison with the standard group, the EAM-guided group had a significant shorter total fluoroscopic time (FT) (1.45 ± 0.58 vs. 12.36 ± 5.46 min, *p* < 0.01) and His lead fluoroscopic time (HL-FT) (0.84 ± 0.56 vs. 9.27 ± 5.44 min, *p* < 0.01), while lower total fluoroscopic dose (3.13 ± 1.24 vs. 25.38 ± 11.15 mGy, *p* < 0.01) and His lead fluoroscopic dose (1.85 ± 1.17 vs. 19.06 ± 11.03 mGy, *p* < 0.01). No significant differences were observed in paced QRS duration and pacing parameters between the two groups. During a 3-month follow-up, one patient had a capture threshold increased >1 V/1.0 ms in the standard group, while no other complications were recorded in either group.

**Conclusion:** The KODEX-EPD system could facilitate HBP implantation with significantly reduced FT and dose without compromising the procedural time.

## Introduction

The traditional right ventricular pacing (RVP) is associated with a higher risk of atrial fibrillation and heart failure and is regarded as a non-physiological pacing modality (1). His bundle pacing (HBP) maintains synchronous ventricular activation by direct stimulation of the His-Purkinje conduction system, which avoids the deleterious effects in RVP, and is considered as an alternative choice in patients who need frequent ventricular pacing (2). However, locating the His bundle (HB) region can be challenging and time-consuming, resulting in significantly higher fluoroscopic exposure compared to traditional RVP (3). In our previous studies, we found that the procedural and fluoroscopic time (FT) could be shortened by a contrast injection visualization technique, however, the overall FT was still relatively longer (4, 5). The higher fluoroscopic exposure can cause damage to both the patients and operators (6). A three-dimensional (3D) electroanatomical mapping (EAM) system has been applied as an efficient way to achieve zero fluoroscopic visualization of the HB region (7). A 3D anatomical image obtained by the EAM system can provide a reliable anatomical reference for searching the HB region with significantly reduced fluoroscopic exposure. Recent studies showed the feasibility of EAM-guided HBP implantation by using the Ensite NavX (St. Jude Medical, St. Paul, MN, USA) or CARTO (Biosense Webster Inc, Irvine, CA, USA) mapping system (8–10).

A KODEX-EPD cardiac imaging and navigation system (EPD Solutions, Philips, Best, The Netherlands) is a novel imaging system that uses dielectric imaging to acquire and display high-resolution anatomical images (11, 12). This system distinguishes materials and generates images based on their different dielectric properties, which is determined by their conductivity and permittivity with respect to the frequency of the electrical field. Compared to the traditional impedance-based systems that use single frequency electrical potential measurements, this dielectric-based system measures conductivity and permittivity at multiple frequencies to determine the dielectric dispersion pattern, which is subsequently used to generate a function that relates the dielectric dispersion to the spatial position in the thorax (Supplementary Table 1 and Supplementary Figure 1). This method is theoretically less susceptible to the inhomogeneities in the body structures and movement of the organs such as heart beat and respiration, which allows for a high spatial resolution. Previous studies showed that this system could provide a computed tomography-like image, and the image quality generated by this system was noninferior to CARTO (13, 14).

In our center, we previously reported a case of HBP implantation facilitated by this novel KODEX-EPD system (15). Since then, we have applied it in a series of patients. In this study, we aimed to assess the feasibility of the KODEX-EPD system-guided HBP implantation in a cohort of patients and compare the procedural outcomes with those who achieved HBP using the conventional fluoroscopy approach.

## Materials and Methods

This is a prospective, randomized study that enrolled 20 patients with bradycardia with an indication for pacing therapy in Fuwai Hospital, Beijing, China from September to December 2020. All patients were grouped by the random number table method and underwent HBP implantation either with the conventional fluoroscopy approach (the standard group) or guided by a novel KODEX-EPD mapping system (the EAM-guided group). The patients were excluded if they: (1) needed implantable cardioverter defibrillator or cardiac resynchronization therapy; (2) were <18-years-old. This study was approved by the Ethics Committee of Fuwai Hospital and written informed consent was obtained from all the patients.

### Implantation Tools

All HBP implantations in this study were performed by using the Select Secure 3830 pacing lead (Medtronic Inc, Minneapolis, MN, USA) and the fixed-curve C315 HIS sheath (Medtronic Inc, Minneapolis, MN, USA).

### Implantation Procedure in the Standard Group

**Figure 1:**
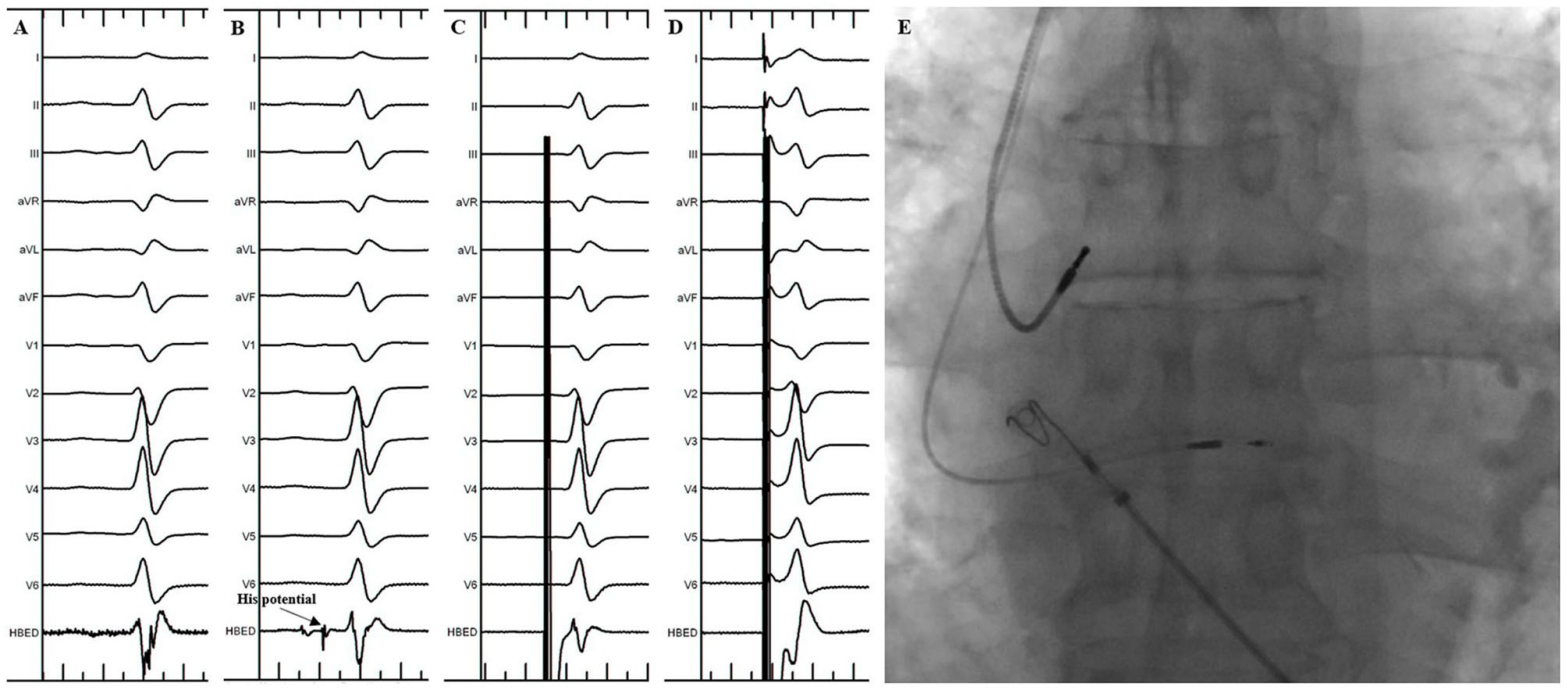
His Bundle Pacing implantation in the standard group. **(A)** Sinus rhythm before the procedure. **(B)** His potential was recorded during the procedure. **(C)** Selective His bundle pacing was confirmed at a lower pacing output. **(D)** Nonselective His bundle pacing was achieved at a higher pacing output. **(E)** Final lead location in the fluoroscopic image.

The procedure of HBP implantation in the standard group was the same as previously described (3). In brief, under the right anterior oblique 30° (RAO 30°) fluoroscopic view, the 3830 pacing lead was placed at the junction of the atrioventricular ring to search the ideal lead deployment site for HBP where the His potential could be recorded or the HB could be captured by unipolar pace mapping (Figure 1). The lead was fixed where the pacing parameters were satisfactory. The unsuccessful HBP were defined as: (1) the HB capture threshold or the correction threshold for bundle branch block (BBB) > 2.5 V/1 ms in three attempts; (2) the total FT was more than 20 min.

### Implantation Procedure in the EAM-Guided Group

**Figure 2:**
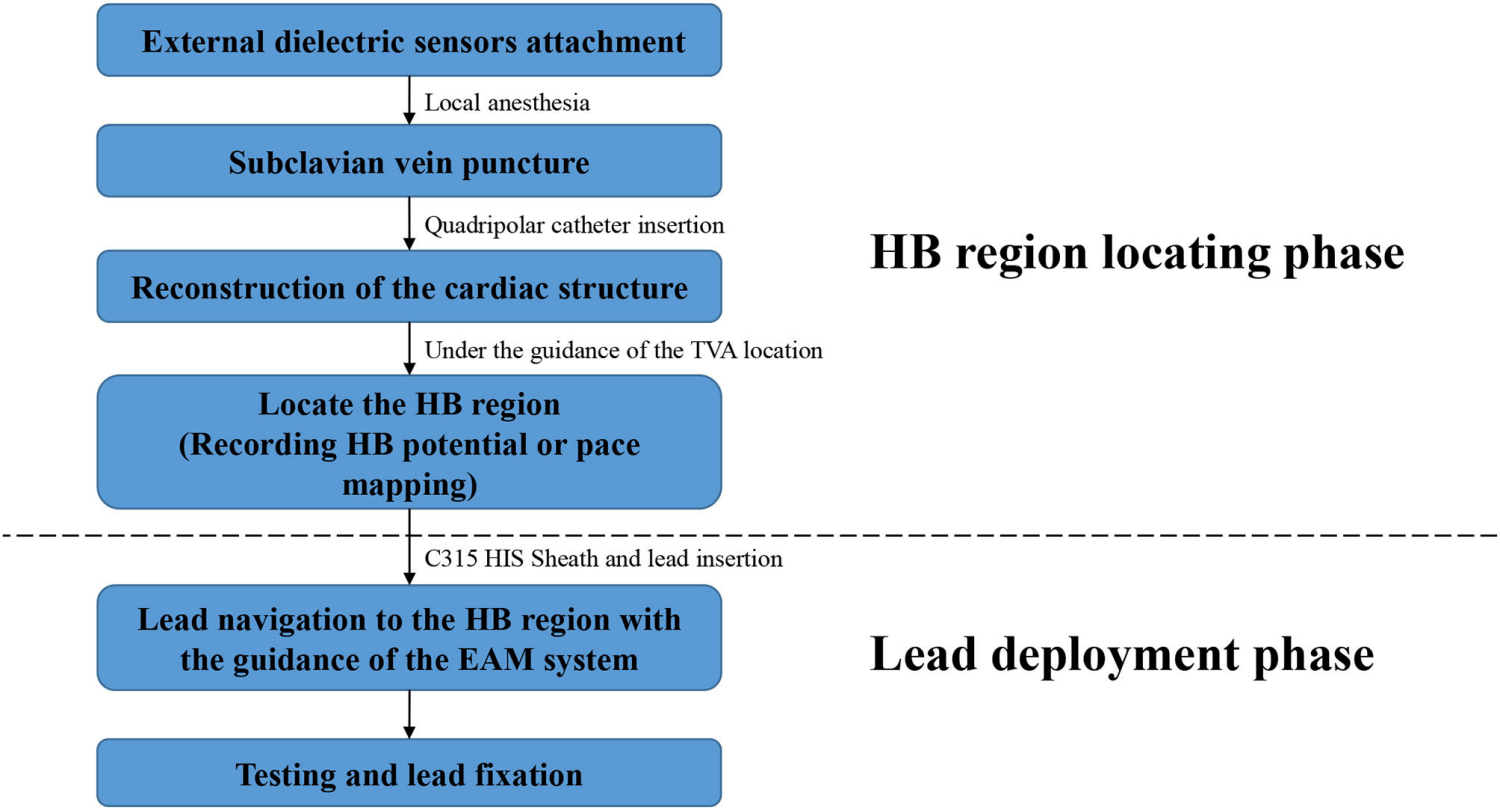
Flowchart of the procedure details in the EAM-guided group. EAM, electroanatomical mapping; HB, His bundle.

**Figure 3:**
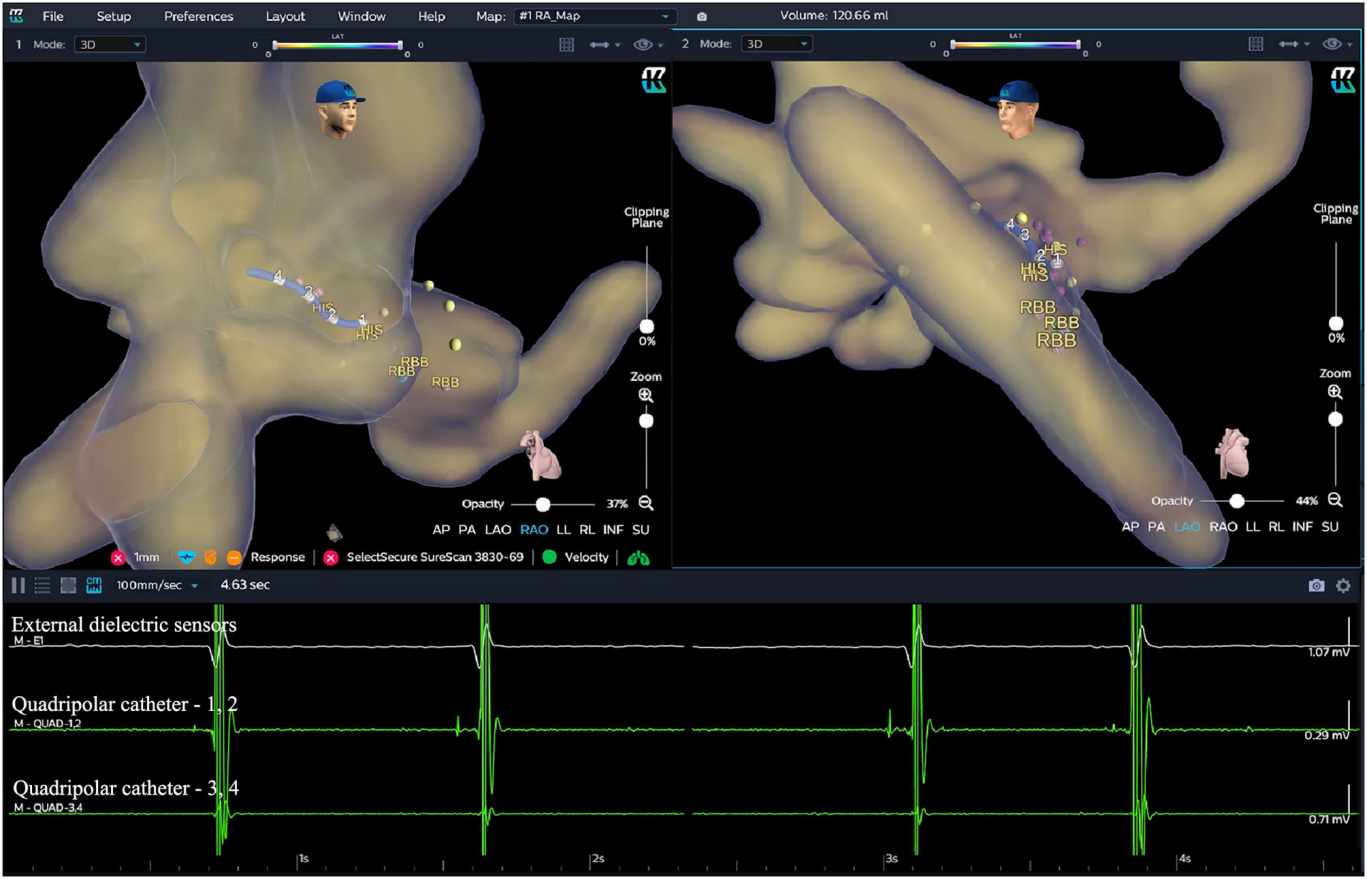
Locating the HB region in the EAM-guided group. Based on the electrical field information, the three-dimensional (3D) cardiac anatomical image was calculated and visualized by moving the catheter without fluoroscopy. The TVA was highlighted (yellow dots in the figure), and the HB region (purple dots in the figure) where the HB potential could be recorded was marked under the guidance of the TVA location. EGMs from top to bottom showed the cardiac signal recorded from the external dielectric sensors, quadripolar catheter 1–2, and quadripolar catheter 3–4, respectively. EAM, electroanatomical mapping; EGM, electrogram; HB, His bundle; RBB, right bundle branch; TVA, tricuspid valve annulus.

**Figure 4:**
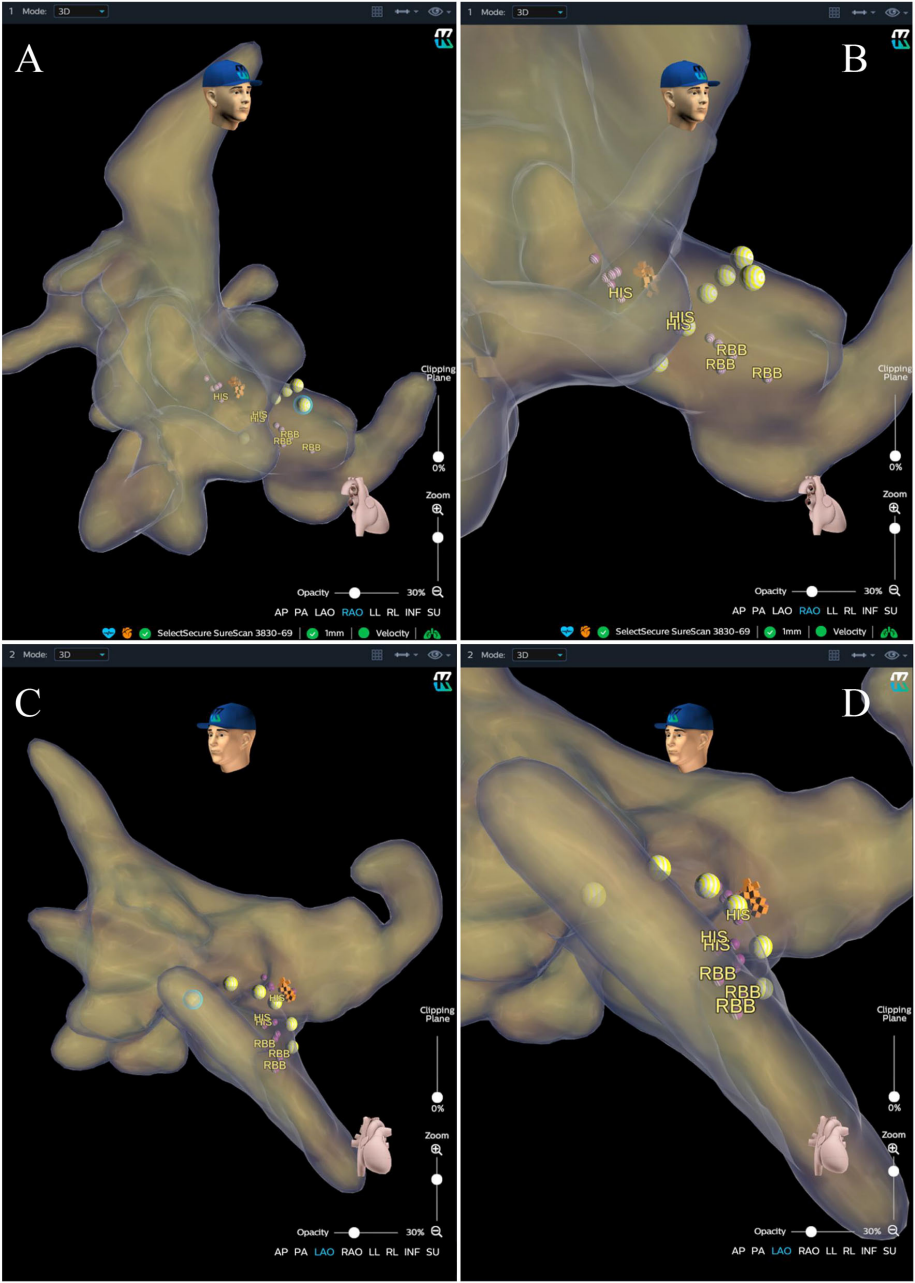
Anatomical image calculated by the KODEX-EPD system. **(A)** Anatomical image in RAO view. **(B)** Enlarged image in RAO view. **(C)** Image in LAO view. **(D)** Enlarged image in LAO view. The purple and orange dots represent the sites where the His bundle potential can be recorded. The yellow dots represent the path of the tricuspid valve annulus. LAO, left anterior oblique; RAO, right anterior oblique; RBB, right bundle branch.

**Figure 5:**
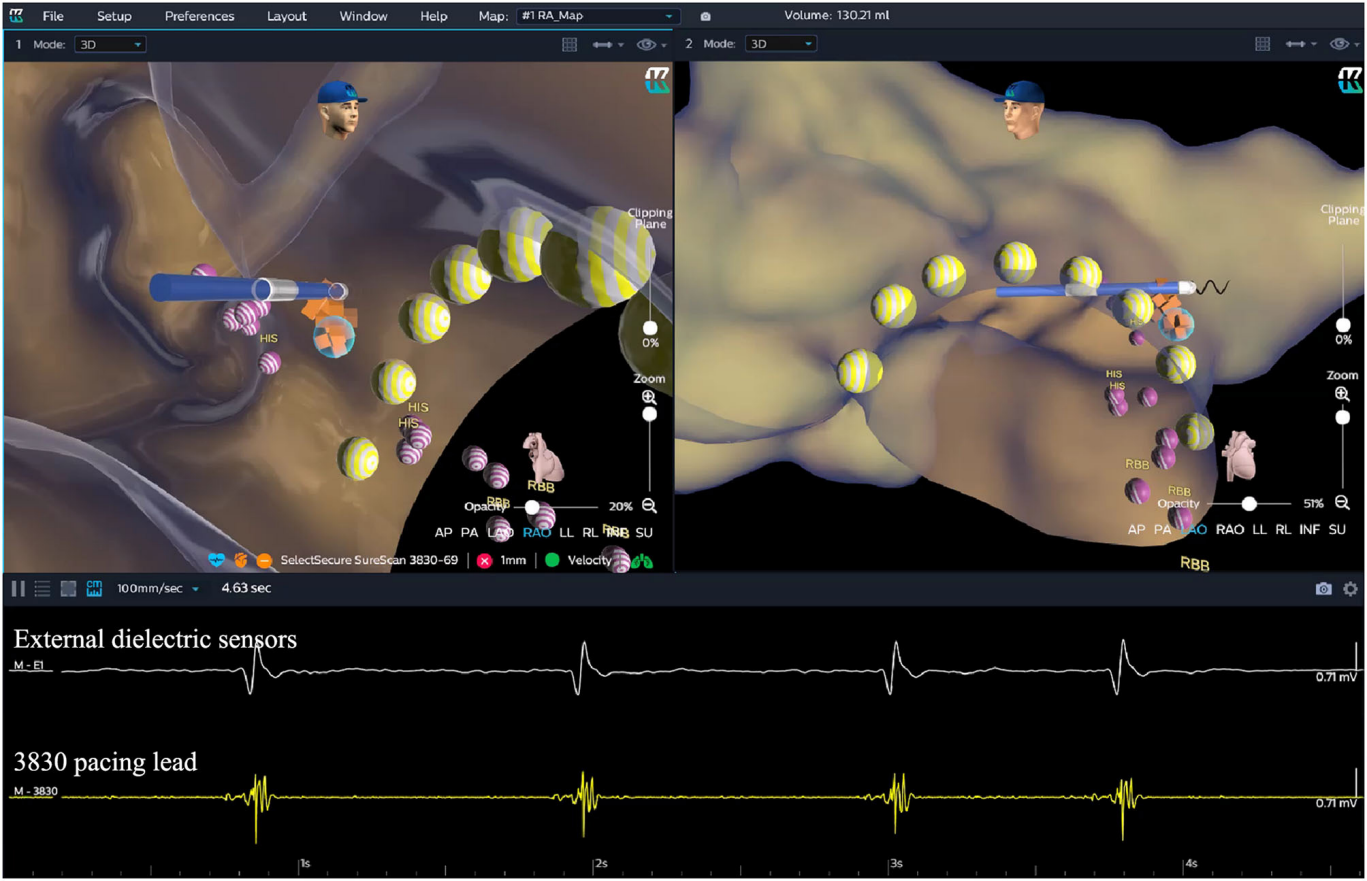
HB lead deployment in the EAM-guided group. After the HB region was successfully marked, the lead was navigated to the HB region under the guidance of the KODEX-EPD system without fluoroscopy. The glass view provided by this system (setting of “opacity”) could give an enhanced perception of the lead location and orientation within the heart. The lead was finally fixed in the target HB region. The yellow dots represent the path of the tricuspid valve annulus. The purple dots represent the sites where the HB potential can be recorded. The orange dots represent the target HB region. EGMs from top to bottom showed the cardiac signal recorded from the external dielectric sensors and the 3830 pacing lead, respectively. EAM, electroanatomical mapping; EGM, electrogram; HB, His bundle; RBB, right bundle branch.

In the EAM-guided group, the procedure of the HBP implantation was similar to the previously reported, and was mainly divided into two steps (Figure 2) (15):

1. Locate the HB Region
  Before the procedure, multiple anisotropic fields were induced by the external dielectric sensors attached to the body surface of the patient. After local anesthesia and the puncture of the left subclavian vein, the quadripolar catheter (Abbott Inc, St Paul, MN, USA) was advanced into the right atrium (RA). Then the KODEX-EPD system was connected to the catheter, which received the sophisticated electrical field information transmitted from the catheter. Based on the electrical field information, the 3D cardiac anatomical image was calculated, and the images of superior vena cava (SVC), inferior vena cava (IVC), RA, tricuspid valve annulus (TVA), right atrial appendage (RAA), and right ventricle (RV) were visualized by roving the catheter within the cardiac chamber without fluoroscopy (Figures 3, 4). Among them, the TVA was highlighted, and the HB region where the HB potential could be recorded was marked under the guidance of the TVA location. The pace mapping (usually 10 mA at 2 ms) was sometimes used to assist in locating the HB region, and the region where the paced QRS morphology showed a selective HBP (S-HBP) or nonselective HBP (NS-HBP) pattern was also marked as the HB region.
2. HB Lead Deployment Guided by the KODEX-EPD System
  Once the HB region was successfully marked, the quadripolar catheter was removed and the C315 HIS sheath was advanced into the RA. Then the 3830 pacing lead was advanced through the sheath with the distal tip exposed. The bipolar sites at the proximal end of the pacing lead were connected to the KODEX-EPD system, and the lead was navigated to the HB region under the guidance of this system (Figure 5). The glass view provided by this system could give an enhanced perception of the lead location and orientation within the heart (Figure 5). The lead was finally fixed in the HB region where the pacing parameters were satisfactory (Figure 5). Similarly, the atrial lead could be placed in the RAA according to the anatomical image shown in this system without fluoroscopy. The criteria of unsuccessful HBP were similar to those in the standard group.

### Data Collection and Follow-Up

Baseline data, such as demographic characteristics, implantation indications, and electrocardiographic measurements were collected at the enrollment. The total FT was the primary endpoint of this study, which was defined as the fluoroscopic duration from delivering the guidewire to the final lead fixation. The His lead fluoroscopic time (HL-FT) was defined as the fluoroscopic duration from advancing the 3830 pacing lead to the His lead fixation. In addition, the procedural time (PT), total fluoroscopic dose (FD), and His lead fluoroscopic dose (HL-FD) were also recorded. The S-HBP was defined as capturing only the HB without adjacent myocardium, which demonstrated an isoelectric interval between the pacing stimulus and the QRS onset. The NS-HBP was defined as capturing both the HB and the adjacent myocardium, in which no isoelectric interval could be observed. The pacing parameters that include capture threshold, R-wave amplitude, and impedance were recorded during the procedure and at 3-month follow-up, and the capture thresholds in patients with BBB recorded in this study were all correction thresholds. Echocardiographic measurements, such as left ventricular ejection fraction (LVEF) and left ventricular end-diastolic dimension (LVEDD) were performed at baseline and 3-month follow-up. The procedure-related complications such as capture threshold increased >1 V/1.0 ms, loss of capture, and lead dislodgement were also tracked during the follow-up.

### Statistical Analysis

Based on the experience at the center, the mean total FT for conventional HBP implantation was estimated at 12 min with a SD of 5 min. By assuming a 75% reduction in total FT by using the EAM system, at least 14 patients required 90% power to detect this mean time reduction with a significance level of α = 0.05. Assuming a 10% withdrawal or implant failure rate, at least 16 patients were needed to be randomized. The continuous data were described as mean ± SD and categorical data were performed as frequencies or percentages. An independent two-sample *t*-test was used to compare the differences between two groups if the data were normally distributed, while Wilcoxon signed-rank test was performed for data that were not normally distributed. Fisher's exact probabilities test was used for categorical variables to determine the differences between groups. A two-sided *P* < 0.05 was considered statistically significant. All the statistical analyses were performed using SPSS Statistics version 22.0 (IBM Corporation, Armonk, NY, USA).

## Results

### Baseline Characteristics

In the study, from September to December 2020, 20 consecutive patients were randomized with 10 patients in each group. As summarized in Table 1, there were no significant differences in baseline characteristics between the two groups. More patients had atrioventricular block (AVB) than sinus node dysfunction (SND) as an indication for pacing therapy in both the groups. The baseline QRS duration in the standard group was similar to that in the EAM-guided group (115.7 ± 20.2 ms vs. 111.2 ± 18.5 ms, *P* = 0.38). Overall, the baseline LVEF and LVEDD were normal in both the groups.

**Table 1:** Baseline characteristics between the two groups.

	**Standard group (***n*** = 10)**	**EAM guided group (***n*** = 10)**	***P*** **-value**
**Demographics**			
Age (years)	57.6 ± 16.2	55.4 ± 15.3	0.76
Male	6 (60.0%)	7 (70.0%)	1.00
**Comorbidities**			
Hypertension	5 (50.0%)	4 (40.0%)	1.00
Diabetes mellitus	2 (20.0%)	2 (20.0%)	1.00
Coronary disease	3 (30.0%)	2 (20.0%)	1.00
**Indications**			1.00
SND	3 (30.0%)	4 (40.0%)	
AVB	7 (70.0%)	6 (60.0%)	
**Baseline ECG**			
QRS duration (ms)	115.7 ± 20.2	111.2 ± 18.5	0.38
LBBB	2 (20.0%)	1 (10.0%)	1.00
RBBB	0 (0.0%)	1 (10.0%)	1.00
**Echocardiography**			
LVEF (%)	60.5 ± 6.1	59.4 ± 5.5	0.68
LVEDD (mm)	51.4 ± 6.4	49.9 ± 3.8	0.53

*AVB, atrioventricular block; ECG, electrocardiogram; LBBB, left bundle branch block; LVEDD, left ventricular end-diastolic diameter; LVEF, left ventricular ejection fraction; RBBB, right bundle branch block; SND, sinus node dysfunction*.

### Procedural Outcomes

The HBP implantation was successfully achieved in nine patients in the standard group and nine patients in the EAM-guided group. One case in the standard group was considered as unsuccessful as it failed to confirm HB capture within 20 min of total FT. In this case, the lead was finally placed at the left bundle branch (LBB) area. One unsuccessful case in the EAM-guided group had advanced His-ventricular conduction disease (HV interval of 80 ms) and left bundle branch block (LBBB), and the pacing output for correcting the conduction block was >2.5 V/1 ms after three lead screw-in attempts at the HB region. Then the lead was further advanced toward the cardiac apex by approximately 2 cm and was placed at the right side of the ventricular septum where the paced morphology showed a “W” pattern in lead V1. After a transient fluoroscopy to confirm the lead perpendicular to the septum, the lead was screwed deep into the left side of the interventricular septum and the paced morphology was carefully monitored to confirm LBB capture (16). Finally, left bundle branch pacing (LBBP) was achieved with a paced QRS duration of 120 ms and a peak left ventricular activation time of 70 ms.

### Procedural Details in the EAM-Guided Group

The procedural details for individual patients in the EAM-guided group are listed in Table 2. As listed in Table 2, nine (90.0%) patients successfully underwent HBP implantation with the guidance of the KODEX-EPD system. Among them, S-HBP was achieved in four (44.4%) patients. The total FT was <2 min in each of the successful patients, and the HL-FT was <1 min in eight of nine patients. In addition, the total FD was <3 mGy in five of nine successful patients, and the HL-FD was <2 mGy in most of the patients (seven of nine).

**Table 2:** EAM-guided group case list.

**Case No**.	**Pacemaker indication**	**Baseline QRSd (ms)/Morphology**	**Capture type**	**Paced QRSd (ms)**	**PT (min)**	**Total FT (min)**	**HL-FT (min)**	**Total FD (mGy)**	**HL-FD (mGy)**
1	AVB	110	S-HBP	110	113	1.8	1.2	3.7	2.5
2	SND	104	S-HBP	104	92	1.1	0.6	2.3	1.3
3	AVB	96	S-HBP	96	78	1.2	0.5	2.4	1.0
4	AVB	106	NS-HBP	132	89	1.5	0.9	3.2	2.0
5	AVB	148/LBBB	Failed, LBBP	120	94	2.9	2.3	6.3	4.9
6	SND	102	NS-HBP	142	63	1.5	0.6	3.2	1.3
7	SND	104	S-HBP	104	77	1.2	0.7	2.7	1.6
8	SND	88	NS-HBP	130	104	1.4	0.7	3.2	1.7
9	AVB	138/RBBB	NS-HBP	134	83	0.9	0.4	2.0	1.0
10	AVB	116	NS-HBP	136	72	1.0	0.5	2.3	1.2

*AVB, atrioventricular block; EAM, electroanatomical mapping; FD, fluoroscopic dose; FT, fluoroscopic time; HL-FD, His lead fluoroscopic dose; HL-FT, His lead fluoroscopic time; LBBB, left bundle branch block; LBBP, left bundle branch pacing; NS-HBP, nonselective His bundle pacing; PT, procedural time; QRSd, QRS duration; RBBB, right bundle branch block; S-HBP, selective His bundle pacing; SND, sinus node dysfunction*.

### Procedural Details and Outcomes Between Two Groups

As shown in Table 3, there were no significant differences in PT between the EAM-guided group vs. the standard group (85.40 ± 22.34 vs. 86.50 ± 15.05 min, *p* = 0.90). Compared to the standard group, the EAM-guided group had a significant shorter total FT (1.45 ± 0.58 vs. 12.36 ± 5.46 min, *p* < 0.01) and HL-FT (0.84 ± 0.56 vs. 9.27 ± 5.44 min, *p* < 0.01). In addition, the total FD (3.13 ± 1.24 vs. 25.38 ± 11.15 mGy, *p* < 0.01) and the HL-FD (1.85 ± 1.17 vs. 19.06 ± 11.03 mGy, *p* < 0.01) in the EAM-guided group were significantly lower as compared to those in the standard group.

**Table 3:** Implantation and follow-up results.

	**Standard group (***n*** = 10)**	**EAM guided group (***n*** = 10)**	***P*** **-value**
Successful HBP	9 (90.0%)	9 (90.0%)	1.00
Dual chamber pacemaker	10 (100.00%)	10 (100.00%)	*N/A*
Procedural details*			
PT (min)	85.40 ± 22.34	86.50 ± 15.05	0.90
Total FT (min)	12.36 ± 5.46	1.45 ± 0.58	<0.01
HL-FT (min)	9.27 ± 5.44	0.84 ± 0.56	<0.01
Total FD (mGy)	25.38 ± 11.15	3.13 ± 1.24	<0.01
HL-FD (mGy)	19.06 ± 11.03	1.85 ± 1.17	<0.01
Paced QRSd (ms)	123.33 ± 20.10	120.89 ± 17.18	0.79
**Pacing parameters**			
Capture threshold (V/1 ms)	1.19 ± 0.35	1.10 ± 0.42	0.63
R-wave amplitude (mV)	5.27 ± 2.30	5.23 ± 1.99	0.97
Impedance (Ω)	576.67 ± 98.08	556.11 ± 98.67	0.66
**Parameters at follow-up**			
Capture threshold (V/1 ms)	1.29 ± 0.32	1.10 ± 0.29	0.21
R-wave amplitude (mV)	5.51 ± 2.84	5.53 ± 2.03	0.93
Impedance (Ω)	489.44 ± 67.77	479.22 ± 72.91	0.76
**Echocardiography at follow-up**			
LVEF (%)	62.00 ± 4.85	60.22 ± 4.41	0.30
LVEDD (mm)	51.11 ± 6.35	49.67 ± 3.12	0.55

*EAM, electroanatomical mapping; FD, fluoroscopic dose; FT, fluoroscopic time; HBP, His bundle pacing; HL-FD, His lead fluoroscopic dose; HL-FT, His lead fluoroscopic time; PT, procedural time; QRSd, QRS duration; LVEDD, left ventricular end-diastolic diameter; LVEF, left ventricular ejection fraction*.

* *Including patients who failed to achieve HBP*.

For patients who had successfully achieved HBP implantation, no significant differences were observed in paced QRS duration between the two groups (the EAM-guided group vs. the standard group: 120.89 ± 17.18 vs. 123.33 ± 20.10 ms, *p* = 0.79). The pacing parameters such as capture threshold (1.10 ± 0.42 vs. 1.19 ± 0.35 V/1 ms, *p* = 0.63), R-wave amplitude (5.23 ± 1.99 vs. 5.27 ± 2.30 mV, *p* = 0.97), and impedance (556.11 ± 98.67 vs. 576.67 ± 98.08 Ω*, p* = 0.66) were similar between the EAM-guided group vs. the standard group.

### Three-Month Follow-Up

During the 3-month follow-up, no significant differences were observed in the pacing parameters (capture threshold, R-wave amplitude, and impedance) and echocardiographic measurements between the two groups (Table 3). One patient in the standard group had a capture threshold increased >1 V/1.0 ms (from 1.1 V/1 ms to 2.3 V/1 ms), and no additional intervention was undertaken. No other procedure-related complications were recorded in both the groups.

## Discussion

In this study, we evaluated a novel KODEX-EPD mapping system that could develop a 3D anatomical image to help in locating the target HB region. The main findings of this study were: (1) the KODEX-EPD system could facilitate HBP implantation with a similar success rate compared to the standard approach; (2) the fluoroscopic time and dose in the EAM-guided group were significantly lower than those in the standard group, while the procedural time was not prolonged; (3) the pacing parameters were similar between the two groups.

His bundle pacing is considered as a physiological pacing modality in patients who need frequent ventricular pacing (17). However, locating the HB region can be challenging and time-consuming due to the small volume of the HB, which results in a significantly increased fluoroscopic exposure compared to the traditional RVP implantation (3). Those effects might increase the risk of genetic transformation or cancer to both the patients and operators (6). The EAM system, which is mainly used in the ablation procedure, is a common tool for zero fluoroscopic visualization of the cardiac structure, and can significantly reduce the radiation exposure of the interventional therapy (7). In addition, compared with the two-dimensional (2D) image obtained by the fluoroscopic image, the 3D anatomical image provided by the EAM system is more efficient in guiding the lead deployment in the pacemaker implantation. The previous studies showed the feasibility of the EAM-guided lead deployment for HBP implantation (8–10). In those studies, they evaluated two conventional EAM systems that include the Ensite NavX and CARTO mapping system.

**Figure 6:**
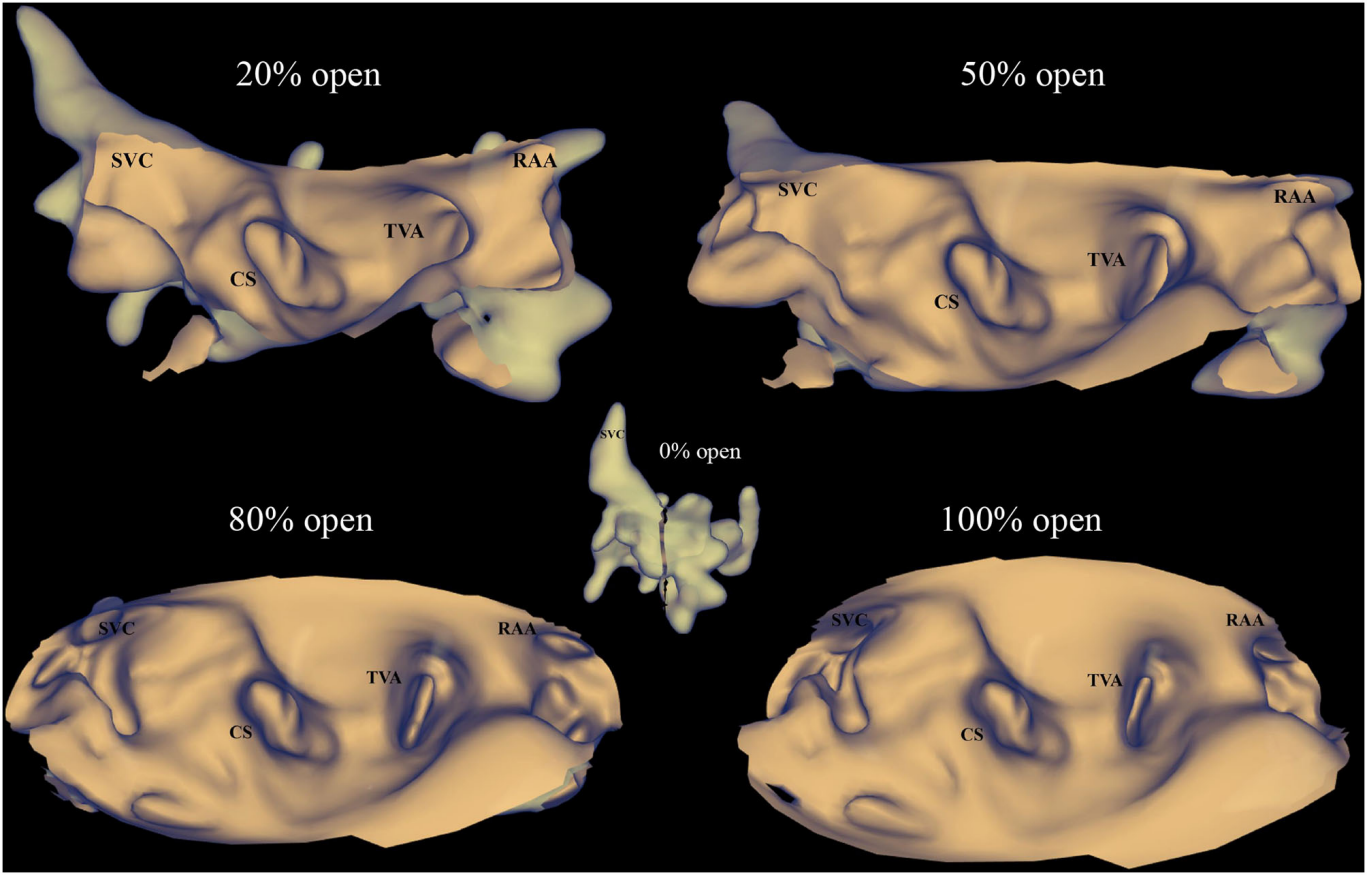
PANO view of the KODEX-EPD system. The anatomical image in the KODEX system can be displayed as a novel panoramic (PANO) view, which transforms the 3D cardiac structure into a virtual two-dimensional (2D) panoramic picture. This view allows all the anatomically relevant structures to be seen in one view. The percentage of open (0% open to 100% open) represents the degree of transformation from a 3D image to a flattened image, 0% open means the image is still shown as a 3D cardiac structure, while 100% open means the full unfolding of the 3D cardiac structure. CS, coronary sinus; RAA, right atrial appendage; SVC, superior vena cava; TVA, tricuspid valve annulus.

KODEX-EPD cardiac imaging and navigation system is a novel wide-band dielectric imaging system, which is designed to acquire and analyze the dielectric energy and use this information to display a high-resolution, real-time 3D image of the cardiac structure (Supplementary Table 1 and Supplementary Figure 1) (11, 12). The anatomical information needed to create the cardiac image is based on the dielectric signals acquired by the external dielectric sensors and the electrophysiological catheter. Even without physical contact with the cardiac wall, the dielectric sensing could show the detailed anatomical information inside of the heart and other structures such as the coronary sinus ostium, pulmonary veins, and atrioventricular valve. The image quality acquired by the KODEX-EPD system is noninferior to the CARTO mapping system as evaluated by the previous studies (13, 14). In addition, during the procedure, the anatomical image can be displayed as a novel panoramic (PANO) view, which transforms the 3D cardiac structure into a virtual 2D panoramic picture (Figure 6). This view allows all the anatomically relevant structures to be seen in one view, which could simplify the catheter or the lead navigation with minimal image maneuvering and make the operator free from the assistance of adjusting the map. Those features can improve the procedural efficiency. The previous studies showed the feasibility of using the KODEX-EPD system in radiofrequency or cryoballoon ablation in clinical practice (14, 18–20).

In this study, we evaluated the feasibility of using this novel mapping system to perform HBP implantation in a cohort of patients. The results showed that the fluoroscopic time and dose were significantly decreased under the guidance of the KODEX-EPD system without compromising the procedural time. To the best of our knowledge, this is the first study that evaluates the feasibility of using this system to guide the HB lead deployment in a cohort of patients with pacing indications. In addition, the KODEX-EPD system can not only facilitate the HBP implantation but also help to guide LBB lead deployment as shown in a patient with failed HBP implantation in the EAM-guided group.

The KODEX-EPD system is compatible with most of the catheters and the leads which are currently used in clinical practice, such as the 3830 pacing lead which is commonly used in His-Purkinje conduction system pacing. In our previous case study, in order to avoid the additional expenses, we did not choose the quadripolar catheter for EAM but directly used the 3830 pacing lead for the whole procedure. In this study, we used the quadripolar catheter instead of the pacing lead for EAM. After the anatomical image was obtained, the HB region could be immediately located according to the anatomical relationship between the HB region and the TVA, and the lead was directly deployed at the HB region. All of the above steps could be achieved with near-zero fluoroscopic visualization, which significantly reduced the fluoroscopic exposure as shown in this study. In order to ensure the safety of the patients, the following steps should be performed under fluoroscopy: (1) delivering the guidewire; (2) removing the sheath; and (3) confirming a proper slack at the lead location (21). These steps added only minimal radiation exposure while the total fluoroscopic time and dose in the EAM-guided group were significantly decreased compared to the standard group. This EAM-guided technique is particularly suitable for specific patients, such as pregnant women.

### Limitations

Several limitations should be emphasized in this study. First, it was a single center study with a small sample size. However, this pilot study showed a significant reduction in the fluoroscopic time and dose, suggesting that this novel EAM system could be a novel zero fluoroscopic guidance tool for HBP implantation. Multicenter studies with a larger population are needed to further evaluate other potential merits with this EAM system. In addition, the mapping system and the catheter need additional costs, which could be offset by a significant reduction in fluoroscopic exposure and a potential improvement in procedural efficiency.

## Conclusions

The KODEX-EPD system can facilitate HBP implantation with significant reduced fluoroscopic time and dose. Further studies with larger sample size are needed to evaluate other potential merits with this EAM system.

## Data Availability Statement

The raw data supporting the conclusions of this article will be made available by the authors, without undue reservation.

## Ethics Statement

The studies involving human participants were reviewed and approved by this study was approved by the Ethics Committee of Fuwai Hospital. The patients/participants provided their written informed consent to participate in this study.

## Author Contributions

WH, MT, and SZ contributed to the conception and design of the study. MG, H-xN, and XC performed pacemaker implantation. XL and MG performed data collection and analysis. The first draft of the manuscript was written by WH and XL and all authors commented on the previous versions of the manuscript. All authors read and approved the final manuscript.

## Conflict of Interest

The authors declare that the research was conducted in the absence of any commercial or financial relationships that could be construed as a potential conflict of interest.

## Publisher's Note

All claims expressed in this article are solely those of the authors and do not necessarily represent those of their affiliated organizations, or those of the publisher, the editors and the reviewers. Any product that may be evaluated in this article, or claim that may be made by its manufacturer, is not guaranteed or endorsed by the publisher.
