# Supplementary material for: Novel Wide-Band Dielectric Imaging System Guided Lead Deployment for His Bundle Pacing: A Feasibility Study
Source: Front Cardiovasc Med. 2021 Sep 3;8:712051. doi: 10.3389/fcvm.2021.712051 (PMC8446512; doi:10.3389/fcvm.2021.712051)
Supplement: Supplementary file 1 [file Data_Sheet_1.pdf]

Supplementary Material

1 Supplementary Figures and Tables

1.1 Supplementary Figures

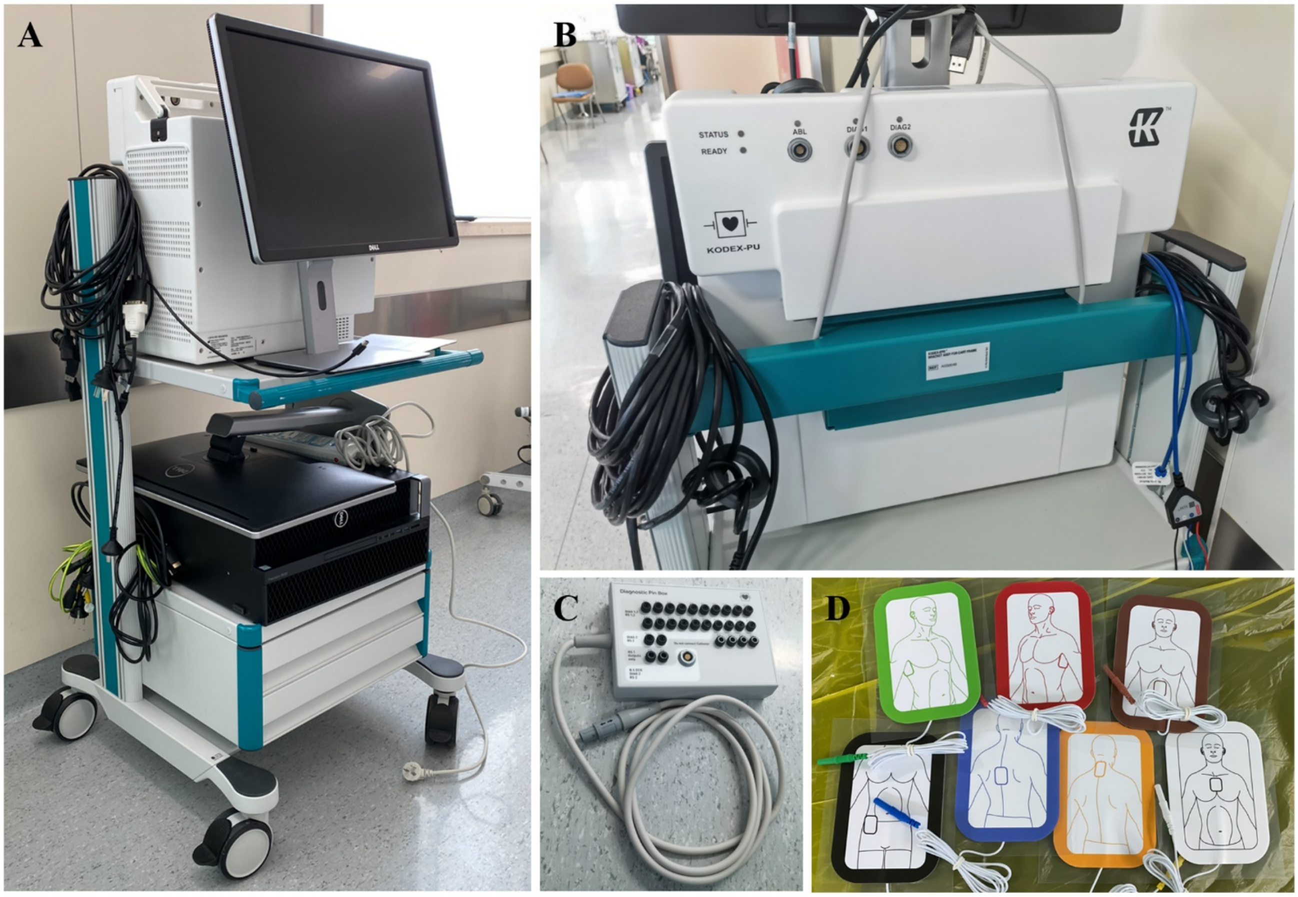

Supplementary Figure 1. Components of the KODEX-EPD system. A. Overview of the KODEX-EPD system. **B.** Processing unit. **C.** Pin box. **D.** Dielectric sensors.

1.2 Supplementary Tables

Supplementary Table 1. Comparison of three EAM systems when used in HBP implantation

**System type**	**Localization and imaging**	**Mapping way**	**Mapping with quadripolar catheter**	**Mapping with 3830 pacing lead**	**Lead navigation with 3830 pacing lead**
KODEX-EPD mapping system	Dielectric properties based, corrected by multi frequency electric field	Tissue contact or non-contact	Yes	Yes	Yes
Ensite NavX mapping system	Electric field based (single frequency electric field)	Tissue contact	Yes	Yes	Yes
CARTO 3 mapping system	Magnetic field based, combined with electric field	Tissue contact	No	No	Yes

EAM, electroanatomical mapping; HBP, His bundle pacing.
